# Dibutyryl Cyclic Adenosine Monophosphate Rescues the Neurons From Degeneration in Stab Wound and Excitotoxic Injury Models

**DOI:** 10.3389/fnins.2018.00546

**Published:** 2018-08-08

**Authors:** Ebtesam M. Abd-El-Basset, Muddanna S. Rao

**Affiliations:** Department of Anatomy, Faculty of Medicine, Kuwait University, Kuwait City, Kuwait

**Keywords:** stab wound injury, hippocampus, BDNF, dBcAMP, astrogliosis

## Abstract

Dibutyryl cyclic adenosine monophosphate (dBcAMP), a cell-permeable synthetic analog of cAMP, has been shown to induce astrogliosis in culture. However, the exact mechanism underlying how dBcAMP exerts its function *in situ* is not clear. The objective of this study was to examine the effects of dBcAMP on astrogliosis and survival of neurons in stab wound and kainic acid models of brain injury. Stab wound was done in cerebral cortex of BALB/c male mice. Kainic acid lesion was induced in hippocampus by injecting 1μl kainic acid into the lateral ventricle. Animals in both models of injury were divided into L+dBcAMP and L+PBS groups and treated with dBcAMP or PBS for 3, 5, and 7 days respectively. The brain sections were stained for Cresyl violet and Fluro jade-B to assess the degenerating neurons. Immunostaining for GFAP and Iba-1 was done for assessing the astrogliosis and microglial response respectively. Expression of GFAP and BDNF levels in the tissue were estimated by Western blotting and ELISA respectively. The results showed a gradual increase in the number of both astrocytes and microglia in both injuries with a significant increase in dBcAMP-treated groups. The number of degenerating neurons significantly decreased in dBcAMP treated groups. In addition, it was found that dBcAMP stimulated the expression of GFAP and BDNF in both stab wound and kainic acid injuries. Treatment with BDNF receptor inhibitor AZ-23, showed an increase in the degenerating neurons suggesting the role of BDNF in neuroprotection. This study indicates that dBcAMP protects neurons from degeneration by enhancing the production of BDNF and may be considered for use as therapeutic agent for treatment of brain injuries.

## Introduction

Traumatic brain injury has a dynamic pathophysiology that includes progressive neuronal loss by apoptosis and necrosis (Rosenfeld et al., 2012) and excitotoxicity. Excitoxicity occurs a results of imbalance between concentrations of glutamate and its agonists (Freire et al., 2016). In cases of central nervous system (CNS) injuries, astrocytes begin to proliferate and accumulate at the margin of the injury forming a layer that interfaces between the injury and intact tissues, which is known as the glial scar or reactive astrogliosis (McGraw et al., 2001). It has been suggested that the scar inhibits axon/nerve regeneration by acting as a physical barrier that axon cannot penetrate (McGraw et al., 2001; Wanner et al., 2008). However, direct evidence for such a role has yet to be definitively shown. Meanwhile, the formation of the glial scar can also be beneficial in isolating the injured region and in preventing the spread of the inflammatory response to adjacent intact neuronal tissue (Sofroniew, 2005; Rolls et al., 2009) thereby helping to limit further expansion of tissue degeneration. The changes that occur during the reactive astrogliosis reflect alterations in the functional activity of astrocytes. The synthesis of many growth factors, cytokines, and neuropeptides that have been turned on in reactive astrocytes suggest that the astrocytic reaction may play an important role in neuronal regeneration (Faulkner et al., 2004; John et al., 2005; Rolls et al., 2009; Colangelo et al., 2014).

Following brain injury activation of microglia and neuroinflammation are implicated in progressive tissue damage. Inhibition of activated microglia following traumatic injury and ischemic lesion using anti-inflammatory indomethacin and microcycline induces transient neuroprotection (Bye et al., 2007; Lopes et al., 2016).

Dibutyryl cyclic adenosine monophosphate (dBcAMP) is a cell-permeable synthetic analog of cyclic adenosine monophosphate (cAMP). dBcAMP is known to induce astrogliosis in astroglia culture (Fedoroff et al., 1984; Abd-El-Basset et al., 1989; Abd-El-Basset, 2000; Aras et al., 2012). cAMP acts as an intracellular second messenger for neurotransmitters and hormones. Although the elevation of dBcAMP levels was reported to promote the axonal growth and functional recovery after spinal cord injury (Pearse et al., 2004) its mechanism of action is not known. In addition, dBcAMP enhances differentiation and survival of neurons from neuronal stem cells transplanted into injured spinal cord (Zahir et al., 2009; Kim et al., 2011; Mothe et al., 2011) and differentiation of mesenchymal stromal cell into neurons (Aquilera-Castrejon et al., 2017). Sen et al. (2011) demonstrated that dBcAMP stimulates the differentiation of astrocytes and increases their multiple functions that is thought to contribute to neuroprotection in case of an injury stress. One mechanism of this neuroprotection is through increasing the expression of brain derived neurotrophic factor (BDNF) that has important role in the development and maintenance of normal brain functions (Barde et al., 1982; Cohen-Cory et al., 2010; Noble et al., 2011; Adachi et al., 2014). BDNF acts as a neurite outgrowth and elongation factor and enhances the dendritic growth (Wirth et al., 2003; Cheng et al., 2011). BDNF also plays role in synapse formation (Vicario-Abejón et al., 2002) and neuroprotection in hypoxic ischemic brain injury (Chen et al., 2013; Wu et al., 2015). Impairment of BDNF in developing and mature brain is implicated in many neurodegenerative and psychiatric diseases (Gratacòs et al., 2007; Zuccato and Cattaneo, 2009; Lu et al., 2013; Ninan, 2014).

The objective of the present experiment was to examine the effects of dBcAMP on gliosis, survival of neurons and its mechanism of action in stab wound and excitotoxic lesion (kainic acid lesion) models of brain injury. Further, to study the effects of dBcAMP on microglial reaction and levels of BDNF in both brain injury models.

## Materials and methods

### Animals

BALB/c male mice were used in the present study (*N* = 324). Mice were maintained in the Animal Resources Centre, Faculty of Medicine, Kuwait University. Mice were fed with food and water *ad-libitum*. Animals were maintained in 12:12 dark: light cycle and room temperature was maintained at 25 ± 2°C. The animal surgery, treatment protocol and maintenance were according to the approved protocol of the Institutional Animal Care and Use Committee of Kuwait University, which follows the recommendations of NIH Guidelines and the Guide for the Care and Use of Laboratory animals. All efforts were made to minimize the number of animals used and their suffering.

### Stab wound model of brain injury

A stab wound was done on the cerebral cortex of 2 months old BALB/c male mice as described earlier (Abd-El-Basset et al., 1989). Briefly, mice were anaesthetized with ketamine (40mg/kg)-xylocane (5mg/kg) mixture (Sigma Chemicals, St. Louis, USA), a stab-wound was made by inserting 21-gauge sterile needle into right frontal cerebral cortex (3 mm depth from skull surface) and 2 mm to the right side of the midline. Animals were divided into two sub-groups: dBcAMP group: mice in this group were treated with dBcAMP (ip, 50 mg/kg, Sigma Chemicals, St. Louis, USA) every day for 3, 5, and 7 days; and control group: mice in this group were treated with PBS for 3, 5, and 7 days. Mice in all groups were euthanized with CO2, perfused with cold PBS on 3rd, 5th, 7th post injury days and fresh tissues around the wound (5mm^3^) was collected for analysis of GFAP content and BDNF level by Western blot and ELISA methods respectively. Additional mice in each group were perfused with saline followed by 4% paraformaldehyde for morphological study by Cresyl violet staining, histochemical staining (Flourojade-B) for neurodegeneration (Schmued and Hopkins, 2000) and immunostaining for astrocytes (GFAP) and microglia (Iba1). Six mice were used in each sub-group. Three independent experiments were done. A total of 108 mice were used for this experiment (Table 1).

**Table 1:** Number for mice in different groups used for morphological and biochemical studies at 3rd, 5th, and 7th day in stab wound model of brain injury.

**Groups**	**Morphological study (CV, FJB, GFAP, Iba1 Staining)**			**Biochemical study [Western Blot (GFAP)** + **ELISA (BDNF)]**			**Total**
	**3rd Day**	**5th Day**	**7th Day**	**3rd Day**	**5th Day**	**7th Day**	
L+PBS	6	6	6	6+6	6+6	6+6	54
L+dBcAMP	6	6	6	6+6	6+6	6+6	54
						**Grand total**	108

*(CV-Cresyl violet staining, FJB-Fluoro-Jade B staining, GFAP-Glial fibrillary acidic protein immunostaining, Iba1-Immunostaing for microglia, BDNF-Brain derived neurotrophic factor*.

### Excitotoxic lesion [intracerebro-ventricular kainic acid (ICV-KA)] model of brain injury

In excitotoxic lesion (kainic acid lesion) model of brain injury/lesion, a lesion was induced in the right hippocampus of 4 months BALB/c mice by injecting 1μl kainic acid (0.25 μg) into the lateral ventricle. Mice were anesthetized with ketamine (40mg/kg)-xylocane (5mg/kg) mixture. Mice were held in the stereotaxic apparatus. Skull surface was exposed by a midline skin incision. A burr hole was drilled with a dental drill (1.5 mm behind the bregma and 2 mm to the right side of the midline following mouse brain stereotaxic atlas Franklin and Paxinos, 2008. One microliter of kainic acid (KA, 0.25μg/μl sterile saline, Sigma Chemicals, St. Louis, USA) was injected into right lateral ventricle with a Hamilton syringe fitted with 26G needle, which was inserted through the bur hole to a depth of 2.5mm from the skull surface. KA injection was done slowly over a period of 10 min. Needle was held in the same position for 20 min, before withdrawing to prevent the back-flow of injected KA. Skin was statured. These mice were divided into L+dBcAMP and L+PBS groups and treated with dBcAMP (ip, 50mg/kg) or PBS for 3, 5, 7 days respectively. First dose of dBcAMP or PBS was given 1h after KA injection. Sham surgery was done on another group of mice, by injection of 1 μl of sterile saline into the lateral ventricle with stereotaxic surgery and injection protocol which was similar to KA injection. Mice were divided into S+dBcAMP and S+PBS groups and treated with dBcAMP (ip, 50mg/kg) or PBS for 3, 5, 7 days respectively. First dose of dBcAMP or PBS was given 1h after sham surgery. At the end of the experiment mice in all groups were sacrificed at 3rd, 5th, and 7th days post injury, brains were dissected and processed for histological, immunostaining and biochemical analysis, as described above under stab wound model of brain injury. Six mice were used in each sub-group. Three independent experiments were done. A total of 216 mice were used for this experiment (Table 2).

**Table 2:** Number for mice in different groups used for morphological and biochemical studies at 3rd, 5th, and 7th day in excitotoxic lesion [Intracerebro-ventricular kainic acid (ICV-KA)] model of brain injury.

**Groups**	**Morphological study (CV, FJB, GFAP, Iba1 Staining)**			**Biochemical study [Western Blot (GFAP), ELISA (BDNF)]**			**Total**
	**3rd Day**	**5th Day**	**7th Day**	**3rd Day**	**5th Day**	**7th Day**	
S+PBS	6	6	6	6+6	6+6	6+6	54
S+dBcAMP	6	6	6	6+6	6+6	6+6	54
L+PBS	6	6	6	6+6	6+6	6+6	54
L+dBcAMP	6	6	6	6+6	6+6	6+6	54
						**Grand total**	216

*CV, Cresyl violet staining; FJB, Fluoro-Jade B staining; GFAP, Glial fibrillary acidic protein immunostaining; Iba1, Immunostaing for microglia; BDNF, Brain derived neurotrophic factor*.

### Tissue fixation and processing for histological and immunostaining

The mice in all groups were euthanized with CO2, at the end of the experiment. Heart was exposed and perfused with 50 mL of heparinized saline followed by 100 mL of 4% paraformaldehyde (in 0.1M Phosphate buffer, pH 7.4). The brain was dissected and post fixed for 48 h in the same fixative. Tissues were cryoprotected in 10, 20, and 30% sucrose solution for 24 h each. Then the tissues were embedded in optimal cutting temperature (OCT) compound (Sigma Chemicals, St. Louis, USA). Thirty micron (30 μm) thick serial, coronal cryostat sections were taken and stored in 24 well culture plate filled with phosphate buffer. Serial brain sections were stained for cresyl violet, Fluoro-Jade-B staining for assessing the degenerating neurons. Further, sections were also immunostained for GFAP and Iba-1 for assessing the astroglyosis and microglial responses respectively.

### Cresyl violet staining

Degenerating cortical neurons in stab wound injury and hippocampal neuronal damage in excitotoxic lesion (ICV-KA) model were assessed by Cresyl violet staining. For this purpose, the brain sections were mounted on gelatinized slides and air dried for overnight. The sections were stained with 0.1% Cresyl violet stain (Sigma Chemicals, St. Louis, USA) for 20 min. Then washed in distilled water and dehydrated in alcohol, then cleared in xylene and mounted with DPX.

### GFAP and Iba1 immunostaining and quantification

Serial, coronal frozen sections were treated with 3% hydrogen peroxide to reduce the endogenous peroxidase activity. The sections were then incubated with polyclonal rabbit anti-GFAP (1:100, Dako, Denmark) or polyclonal rabbit anti-Iba-1(1:500, Abcam, Cambridge, MA, USA) antibodies overnight at 4°C. The sections were incubated with biotinylated anti-rabbit IgG (1:20, Vector Labs, Burlingame, CA, USA) for 1 h, followed by incubation with ExtrAvidin peroxidase (1:20, Sigma Chemicals, St. Louis, USA) for another 1 h. The color was developed using DAB (Vector Labs, Burlingame, CA, USA) as chromogen. Sections were mounted on gelatin coated slides, air dried, dehydrated in ethyl alcohol, cleared in xylene and mounted with DPX. Number of GFAP stained astrocytes and number of Iba-1stained microglia were counted in six randomly selected fields (photographs taken with 40x objective) in each section around the lesion site in stab wound injury and from dentate gyrus and CA3 regions in kainic acid lesion model.

From each mouse six sections, (60 μm apart) were selected for quantification. Finally, number of astrocytes, microglia or degenerating neurons/mm^3^ tissue were calculated for each mouse by using stereological principle as described by Beauquis et al. (2014). Briefly, total number of degenerating neurons, astrocytes and microglia/mm^3^ tissue were quantified by using the data collected from coronal brain sections using the formula: T = N^*^V/t, where N is the numerical cell density, V is the volume of the lesioned tissue (Around the lesion site in stab wound injury, CA3, Dentate gyrus, and Dentate hilus in the excitotoxic injury model) used for quantification and *t* is section thickness (30 μm). The numerical cell density (N) was calculated by measuring the area the section used for quantification with NIS-Elements software (NIS-Elements-D2.20) and total cell counts in six randomly selected fields. The volume (V) was calculated by measuring the area of the section used for quantification (Cortical tissue around the lesion site, CA3, dentate gyrus (dentate gyrus + dentate hilus), dentate hilus region) with NIS-Elements software (NIS-Elements-D2.20) and multiplying by section thickness (30 μm), inter-section distance (60 μm) and number of sections (6). All the brain sections were examined by Olympus BX51 TF upright transmitted light/fluorescence microscope using objective 20X (aperture is 0.50) and 40X (aperture is 0.75).

### Fluoro-Jade B (FJ-B) staining and quantification

Brain sections were mounted on gelatin-coated slides and air-dried overnight at room temperature. Slides were hydrated and treated with 0.06% potassium permanganate (Sigma Chemicals, St. Louis, USA) for 20 min, washed in distilled water and incubated in 0.004% FJ-B (Histo-Chem, Inc. Jefferson, Arkansas, USA) solution in 0.1% acetic acid for 30 min at 25°C. The sections were rinsed in distilled water, dehydrated and mounted with DPX. Number of FJ-B stained degenerating neurons were counted in six randomly selected field/section (Photographs taken with 40x objective) around the lesion site. In excitotoxic lesion model, from each mouse six sections/mice (60 μm apart) were selected for quantification. Number of FJB positive neurons in entire CA3 and dentate hilus region were counted. Finally, mean number of degenerating neurons/mm^3^ were calculated as described above.

### Polyacrylamide gel electrophoresis (PAGE) and immunoblotting

Animals were perfused with 50 mL of cold saline. Tissue around the injury site (5mm^3^) in the stab wound injury or entire hippocampus in excitotoxic lesion, was removed and snap frozen in liquid nitrogen and stored at −80°C until Western Blot analysis. For analysis, tissue was thawed and incubated in ice cold radioimmunoprecipitation assay (RIPA) lysis buffer, with sodium orthovanadate (0.5 mM), and the protease inhibitors, phenylmethanesulfonyl fluoride (PMSF; 1 mM), aprotinin (10 μg/ml), leupeptin (1 μg/ml) for 10 min. Tissue was homogenized in cold, in a tissue homoginiser for 3–5 min, homogenate was centrifuged at 14,000 rpm at 4°C for 5 min to collect the supernatant. The protein concentration in the samples was determined using a spectrophotometer (Bioteck). All samples (75 μg protein/well) were analyzed electrophoretically on a 10% SDS-PAGE gel (Laemmli, 1970). The proteins in the gel were transferred to nitrocellulose membrane (Towbin et al., 1979). After transfer, the membranes were incubated for 1 h with 5% skim milk in Tris-buffered Saline-Tween 20 (TBST). The immunoblots were probed with antibodies rabbit anti-GFAP diluted in 5% skim milk in TBST, and rabbit anti-Glyceradehde-3-phosphate dehydrogenase (GAPDH, Sigma Chemicals, St. Louis, USA) were used as endogenous sample loading control. This was then followed by incubation with affinity-purified goat anti-rabbit conjugated to horse-radish peroxidase (1:100, Sigma, Chemicals, St. Louis, USA). Immunoreactive bands were visualized using an enhanced chemiluminescence system (ECL, Santa Cruz Biotechnology, Inc. Dallas, Texas U.S.A). The difference in the band intensities on exposed films were determined by densitometric scanning. Intensity of bands were quantified in the Image-J image analysis software.

### ELISA for BDNF

Tissue samples were collected as described above (5 mm^3^ tissue around lesion site in the stab wound injury or entire hippocampus in excitotoxic lesion, snap frozen in liquid nitrogen, stored at −80°C) and were weighed immediately to get the wet weight of the samples. Each sample was transferred to 250 μl of ice-cold homogenization buffer and homogenized for 1 min in a tissue homogenizer. Composition of the homogenization buffer was 100mM Tris/HCl, pH 7, 2% bovine serum albumin (BSA), 1M NaCl, 4mM EDTA.Na2, 2% Triton X-100, 0.1% sodium azide and the protease inhibitors (5 μg/mL aprotinin, 0.5 μg/mL antipain, 157 μg/mL benzamidine, 0.1 μg/mL pepstatin A, and 17 μg/mL phenylmethyl-sulphonyl fluoride). The lysate from each sample was centrifuged at 14,000 × g for 30 min at 4°C and the supernatant solutions were collected. The supernatant from each sample was frozen for subsequent measurements of BDNF by using ChemiKine brain derived neurotrophic factor (BDNF) sandwich ELISA kit (Merck Millipore, Billerica MA, USA) following the protocol provided in the kit. Briefly 100 μL of standards or appropriately diluted samples were added into each flat bottom wells, pre-coated with mouse anti-Human BDNF monoclonal antibody and incubated at 4°C overnight on a shaker. Wells were washed three times with 250 μL of diluted wash buffer. Hundred microliter of the diluted biotinylated mouse anti-BDNF monoclonal antibody (1:1000 in sample diluent) was added to each well and incubated at room temperature for 3 h on a shaker. Wells were washed again three times with 250 μL of diluted wash buffer. Hundred microliter of the diluted streptavidin-HRP conjugate solution (1:1000, in sample diluent) was added to each well and incubated at room temperature for 1 h on a shaker. Wells were washed three times with 250 μL of diluted wash buffer. Hundred microliter of TMB/E substrate (3,3′,5,5′-tetramethylbenzidine) was added to each well and incubated at room temperature for 15 min. Reaction was stopped by adding 100 μL of stop solution to each well. The wells were read immediately in a ELISA plate reader at 450 nm. Optical density of standard solution was plotted against known concentration of the standards to get the standard curve. Unknown concentration of the BDNF in the samples was calculated by plotting their OD values into the standard curve.

### Assessment of role of BDNF in neuroprotection

In order to assess the role of BDNF and demonstrate its role in the neuroprotection in the stab wound injury, we used the BDNF receptor inhibitor (Trk kinase inhibitor, AZ-23) to inhibit the activity of BDNF in a separate experiment. Two groups of mice with stab wound injury were treated with dBcAMP (ip, 50 mg/kg) for 4 days. First group of mice were treated orally with AZ-23 (20 mg/kg) every 12 h during these 4 days of dBcAMP treatment (Thress et al., 2009). Second group of mice were treated with the vehicle (DMSO) every 12 h, for 4 days served as vehicle control group. Another group of stab wound injured mice were treated with PBS for 4 days, served as control group. Animals were perfused 10 h after the last AZ-23 administration and processed for Fluoro-Jade B staining. Number of degenerating neurons around the injury site were quantified using same quantification protocol for other experiments.

### Statistical analysis

Number of GFAP stained astrocytes, Iba-1stained microglia and degenerating neurons were counted in six randomly selected fields (photographs taken with 40x objective) in each section around the lesion site in stab wound injury and from dentate gyrus and CA3 regions in kainic acid lesion model. From each mouse six sections, (60 μm apart) were selected for quantification. Six mice were used in each sub-group. Finally, number of astrocytes or microglia or degenerating neurons/mm^3^ tissue were calculated for each mouse by using stereological principle (Beauquis et al., 2014). Three independent experiments were done. The data were expressed as Mean ± SEM and were analyzed by one-way ANOVA, followed by Bonferroni's post-test. *P*-values < 0.05 was considered statistically significant.

## Results

### Effect on neurodegeneration

**Figure 1:**
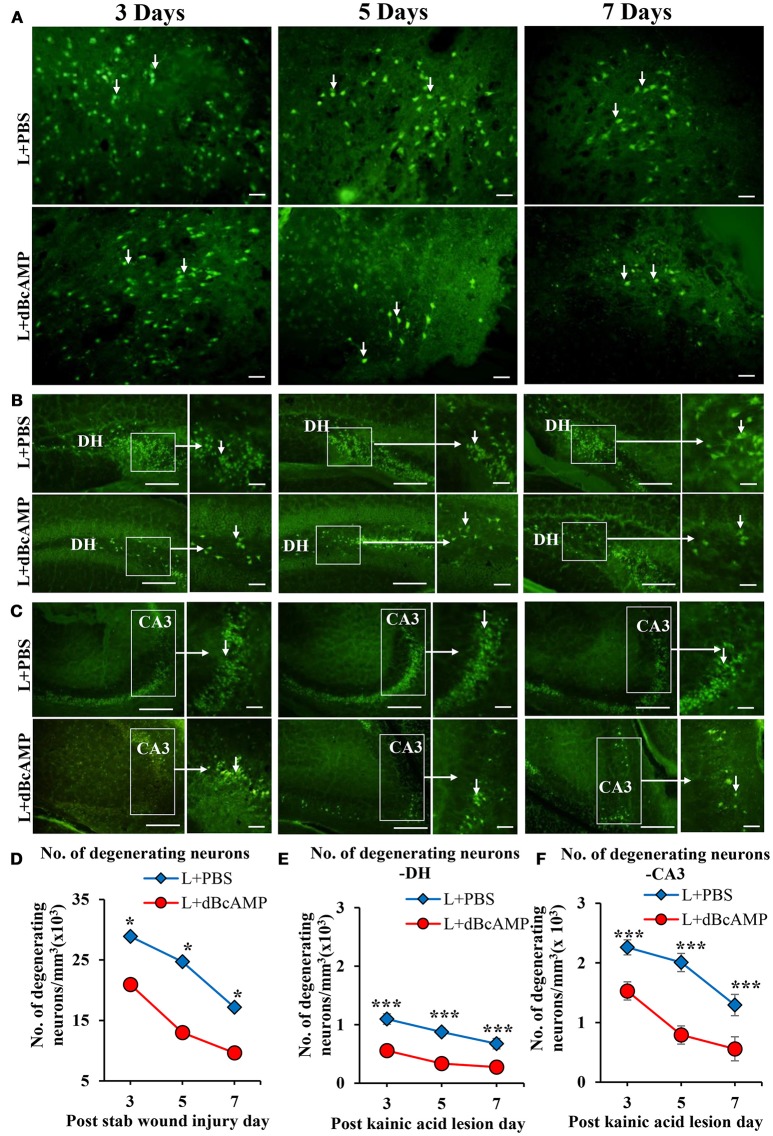
Photomicrograph of Fluoro-Jade B stained sections of the brain, showing degenerating neurons (arrows) in **(A)** Stab wound injury, **(B)** Excitotoxic lesion in the dentate hilus region (DH), **(C)** Excitotoxic lesion in the CA3 region. Note the decreased number of degenerating neurons in dBcAMP treated groups compared to the control groups in both types of injury. Scale bar = 30 μm. Graphs showing the number of degenerating neurons around the stab wound injury site **(D)**, DH **(E)** and CA3 region **(F)**. Note significantly decreased number of degenerating neurons in dBcAMP treated groups compared to the control groups in stab wound injury at all-time points studied (*p < 0.05). Number of degenerating neurons in dBcAMP treated groups in excitotoxic lesioned hippocampus decreased significantly compared to L+PBS groups both in DH and CA3 region at all-time points studied (***p < 0.001).

**Figure 2:**
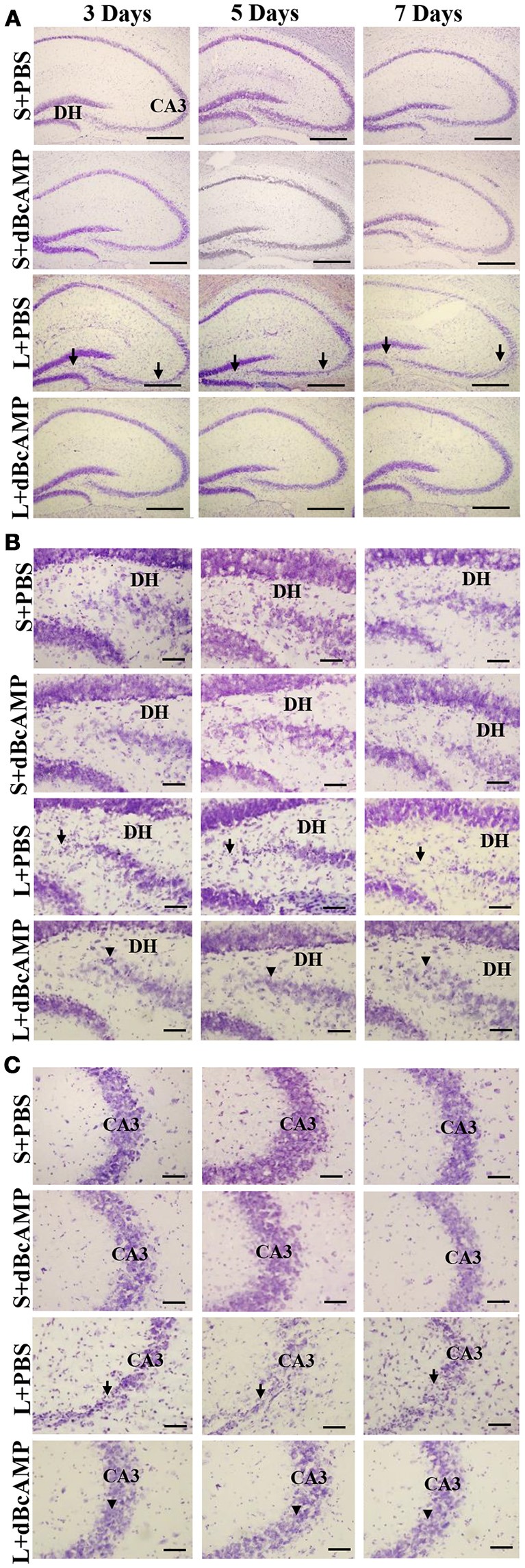
**(A)** Low magnification photomicrograph of the hippocampus stained with Cresyl violet stain to show the excitotoxic lesion in the dentate hilus (DH) and CA3 regions. **(B,C)** High magnification photomicrograph of the dentate hilus region **(B)**, and CA3 **(C)** stained with Cresyl violet stain. Note the noticeable lesion in the CA3 and dentate hilus region in L+PBS groups (arrows), however lesion effect is reduced in the same regions in dBcAMP treated groups (arrowheads). No remarkable changes in sham control (S+PBS) or sham+dBcAMP (S+dBcAMP) treated groups. Scale bar = 120 μm in A and = 60 μm in **B**,**C**.

FJ-B staining showed a significant decrease in the number of degenerating neurons in dBcAMP treated groups, compared to PBS treated groups at 3, 5, and 7 days after stab wound injury (*p* < 0.05, Figures 1A,D) and after excitotoxic lesion, both in dentate hilus and CA3 region (*p* < 0.001, Figures 1B,C,E,F). Cresyl violet staining showed a noticeable excitotoxic lesion in the dentate hilus and CA3 regions in lesioned groups (L+PBS), however lesion effect was reduced in the same regions in dBcAMP treated groups (L+dBcAMP, Figures 2A–C). There were no remarkable changes in sham control (S+PBS) or Sham +dBcAMP treated groups.

### Effect on astrocytes (GFAP)

**Figure 3:**
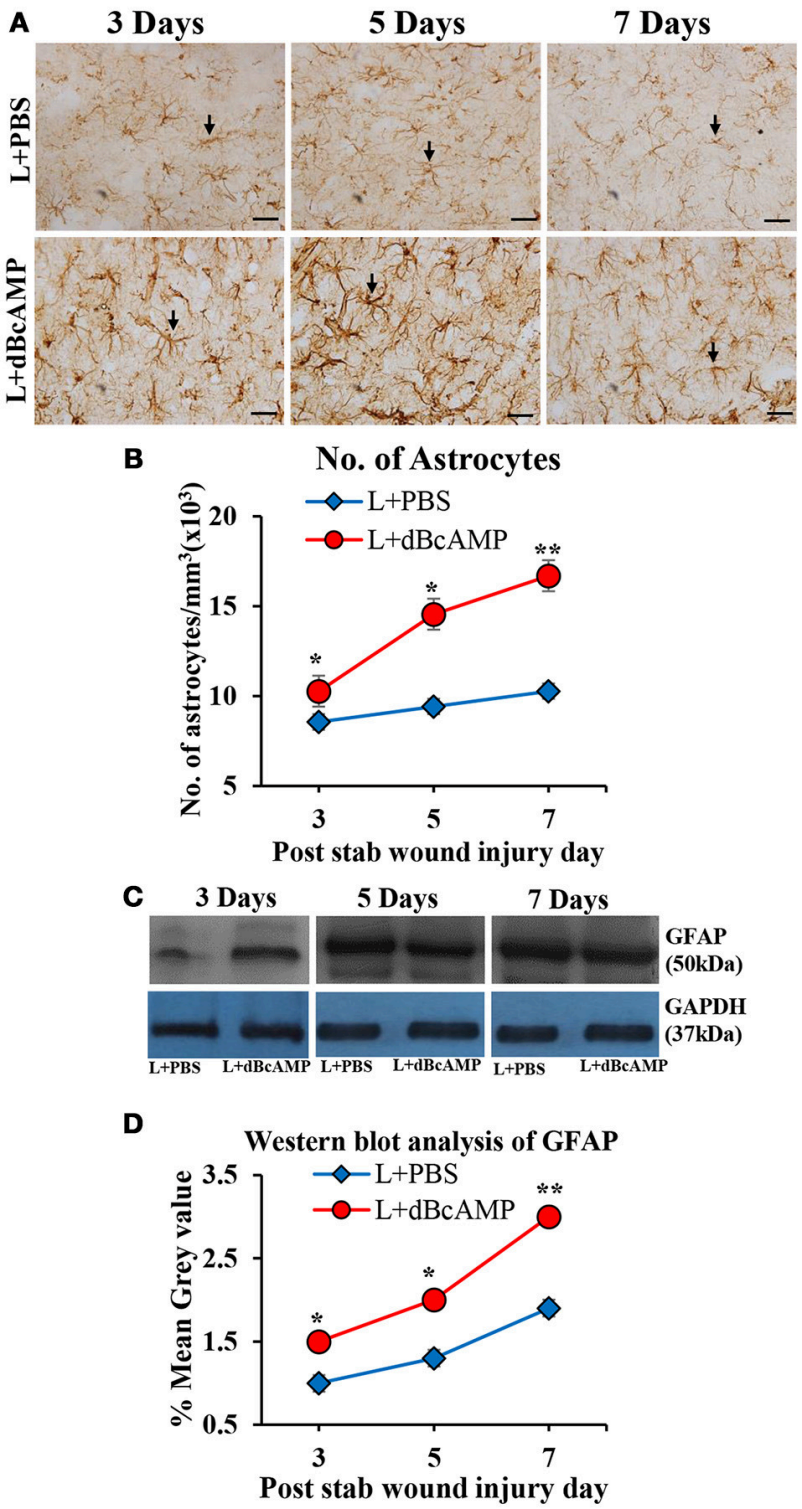
**(A)** Photomicrographs of GFAP immunostained brain sections from the stab wound injury showing the astrocytes (arrows). Note the increase in the number of astrocytes in dBcAMP treated groups compared to the control groups. Scale bar = 30 μm. **(B)** Graph showing the number of astrocytes around the stab wound injury site. Note significantly increased number of astrocytes in dBcAMP treated groups compared to the control groups at all-time points studied (*p < 0.05, **p < 0.01). **(C)** Immunoblotting of GFAP from 3, 5, and 7 days post stab wound injury (dBcAMP treated and control groups) stained with anti-GFAP and anti-GAPDH antibodies. **(D)** Graph showing the mean gray value (GFAP/GAPDH) of the immunoblots. Note significantly increased GFAP content in dBcAMP treated groups compared to the control groups at all-time points of study (*p < 0.05, **p < 0.01).

**Figure 4:**
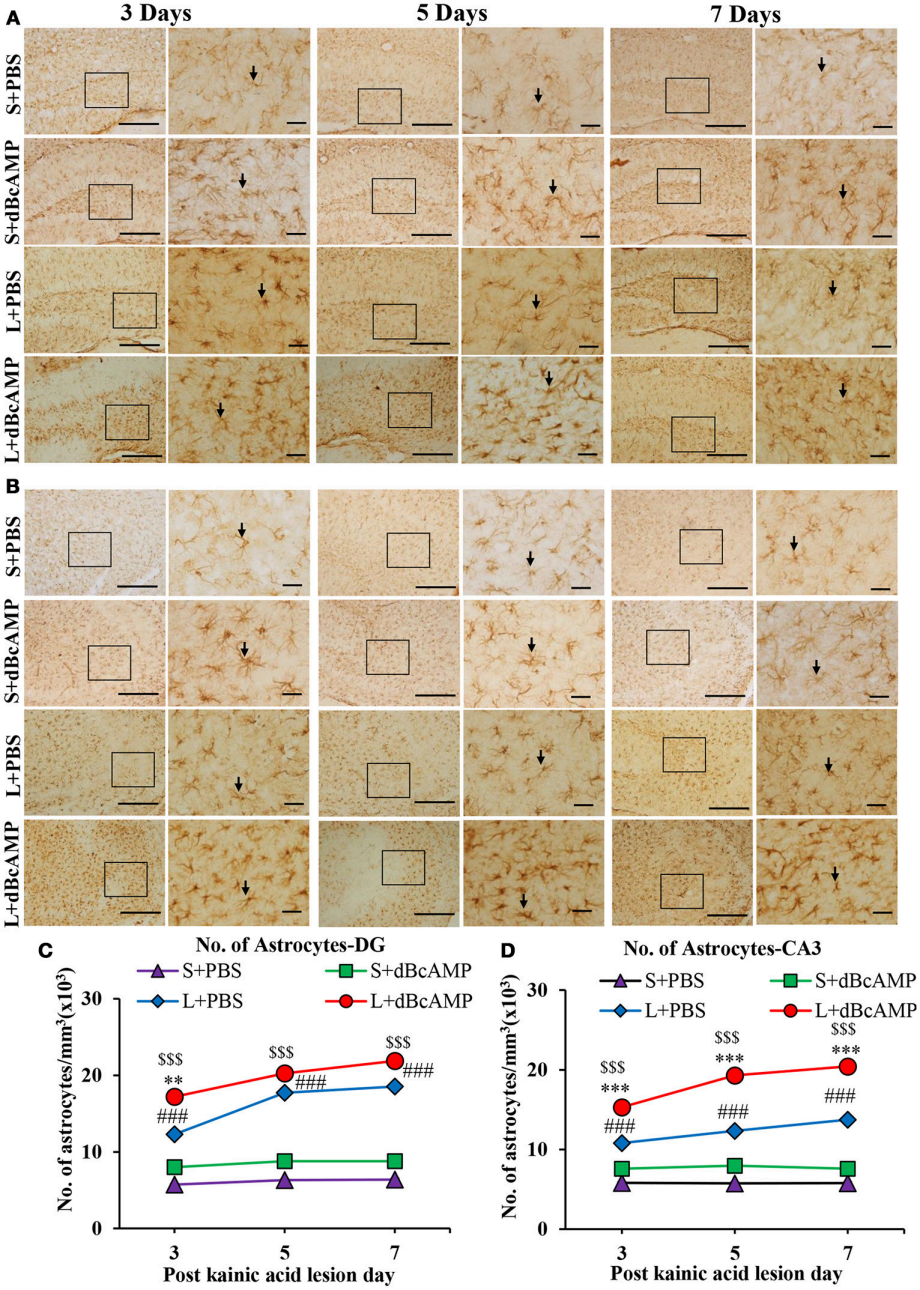
**(A,B)** Photomicrographs of dentate gyrus region **(A)** and CA3 region **(B)** in excitotoxic injury model, immunostained for GFAP. A magnified view of the area indicated by the rectangular box is shown for each time point studied. Note the increased number of astrocytes (arrows) in dBcAMP treated groups compared to L+PBS groups. No remarkable changes in sham control (S+PBS) or sham+dBcAMP treated groups at both regions. Scale bar = 60μm. **(C,D)** Graphs showing the number of astrocytes in dentate gyrus region **(C)** and in CA3 **(D)** and. Note significantly increased number of astrocytes in dBcAMP treated groups compared to L+PBS groups at all-time points studied. (S+PBS vs. L+PBS: ###, *p* < 0.001; S+PBS vs. L+dBcAMP: $$$, *p* < 0.001; L+PBS vs. L+dBcAMP: **p < 0.01; ***p < 0.001). No significant increase in the number of astrocytes in S+PBS and S+ dBcAMP groups.

**Figure 5:**
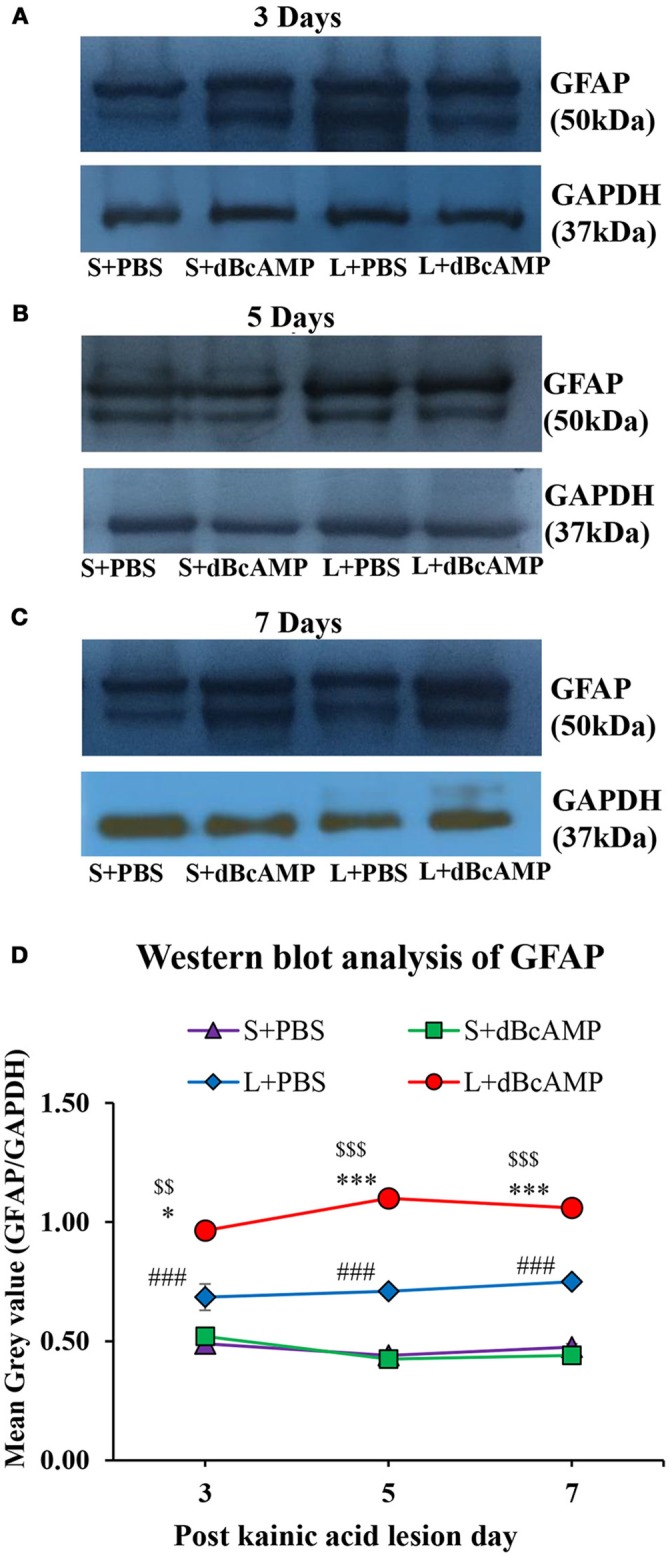
**(A–C)** Immunoblotting of GFAP from 3, 5, and 7 days post excitotoxic injury hippocampal tissue stained with anti-GFAP and anti-GAPDH antibodies. **(D)** Graph showing the mean gray value (GFAP/GAPDH) of the immunoblots. Note significantly increased GFAP content in dBcAMP treated groups compared to control groups at all-time points of study (S+PBS vs. L+PBS: ^*###*^*p* < 0.001; S+PBS vs. L+dBcAMP: ^$$^*p* < 0.01; ^$$$^*p* < 0.001; L+PBS vs. L+dBcAMP: *p < 0.05; ***p < 0.001).

dBcAMP significantly increased the number of astrocytes surrounding the cortical injury at 3, 5 and 7 days post stab wound injury compared to the control groups (*p* < 0.05, 0.01, Figures 3A,B). Western blot analysis for GFAP revealed an increased amount of GFAP expressed by astrocytes in the stab wound injury (Figures 3C,D). Excitotoxic lesion of the hippocampus resulted in significantly increased number of astrocytes in dentate gyrus (DG) and CA3 region in dBcAMP treated groups compared to L+PBS groups at all-time points studied (*p* < 0.05–0.001, Figures 4A–D). The amount of GFAP expressed by astrocytes was increased in excitotoxic lesion as revealed by Western blot analysis for GFAP (Figures 5A–D). There were no significant changes in astrocyte number or GFAP content in sham groups (S+PBS vs. S+ dBcAMP).

### Effect on microglia (Iba-1)

**Figure 6:**
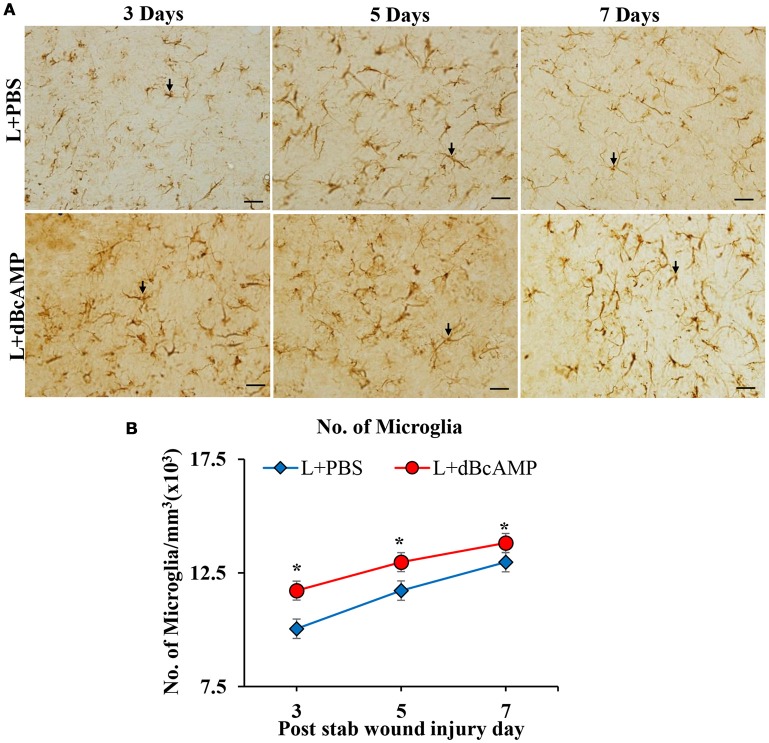
**(A)** Photomicrographs of brain sections of stab wound injury immunostained for microglia with Iba-1 antibody, showing immunopositive microglia (arrows). Note increase in the number of microglia in dBcAMP treated groups compared to the control groups. Scale bar = 30μm. **(B)** Graph showing significant increase in the number of microglia in dBcAMP treated groups compared to the control groups at all-time points studied (*p < 0.05).

**Figure 7:**
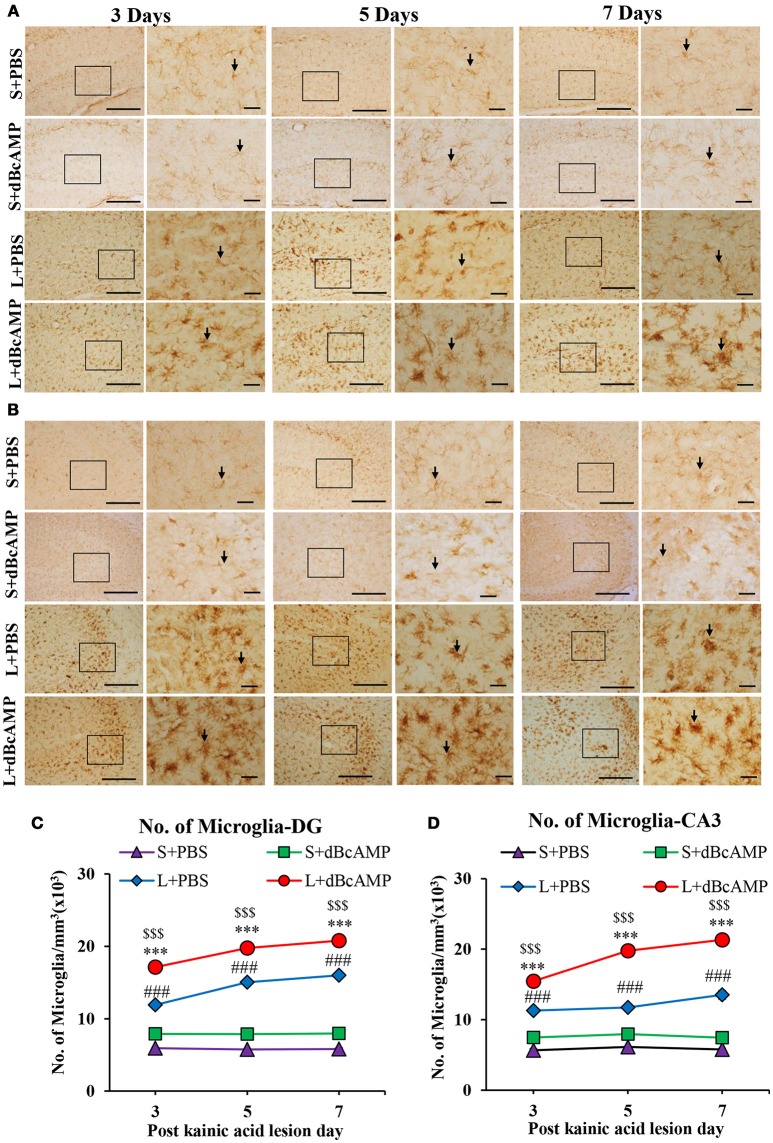
**(A,B)** Photomicrographs of dentate gyrus **(A)** and CA3 region **(B)** in excitotoxic lesioned brain, immunostained for microglia with Iba-1 antibody (arrows). A magnified view of the area indicated by the rectangular box is shown for each time point studied. Note the increased number of microglia in dBcAMP treated groups compared L+PBS groups. No remarkable changes in sham control (S+PBS) or sham + dBcAMP treated groups. Scale bar = 60 μm. **(C,D)** Graphs showing the number of microglia in dentate gyrus (DG) **(C)** and CA3 region **(D)**. Note the number of microglia increased significantly in dBcAMP treated groups compared to L+PBS groups at all-time points studied (S+PBS vs. L+PBS: ###, *p* < 0.001; S+PBS vs. L+dBcAMP: $$$, *p* < 0.001; L+PBS vs. L+dBcAMP: ***p < 0.001). No significant changes of microglia in sham control (S+PBS) or sham + dBcAMP (S+dBcAMP) treated groups.

The number of microglia in dBcAMP treated group was significantly high compared to the control group at all-time points studied in the stab wound injury model (*p* < 0.05, Figures 6A,B). In case of the excitotoxic lesion of the hippocampus (kainic acid lesion) the number of microglia in CA3 and dentate gyrus region was significantly increased in dBcAMP treated groups compared to L+PBS groups at all-time points studied (*p* < 0.001, Figures 7A–D). There were no significant changes in microglial number in sham groups (S+PBS vs. S+ dBcAMP).

### Effect on BDNF level

**Figure 8:**
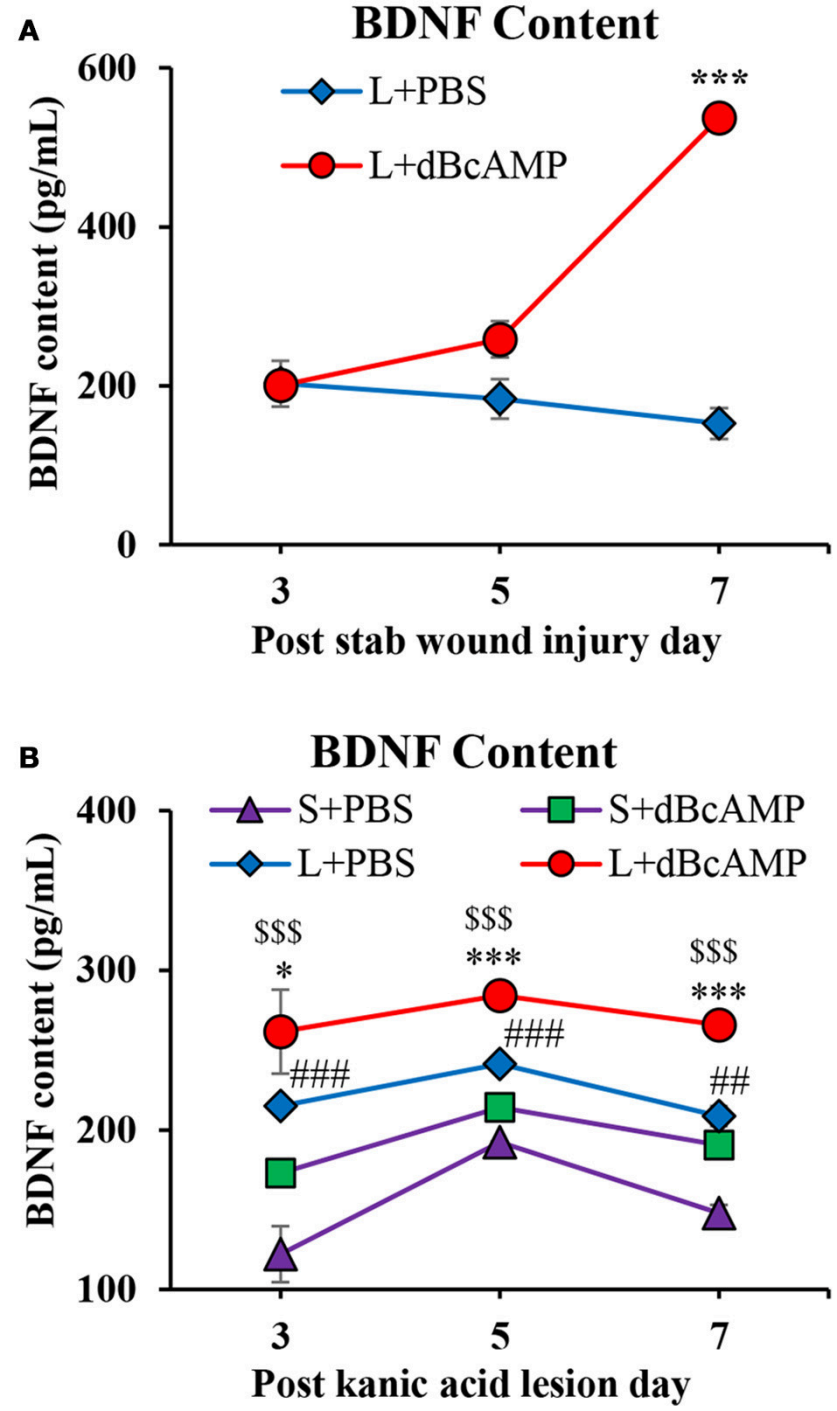
**(A,B)** Graphs showing the concentration of BDNF in tissue from the lesion site in different groups in stab wound injury **(A)** and in the hippocampal tissue in excitotoxic lesion **(B)** Note significantly increased BDNF content in dBcAMP treated groups compared to the control groups on 7th post stab wound injury day (***p < 0.001). BDNF content was significantly increased in dBcAMP treated groups compared to S+PBS and L+PBS groups. (S+PBS vs. L+PBS: ^*##*^*p* < 0.01, ^*###*^*p* < 0.001; S+PBS vs. L+dBcAMP: $$$, *p* < 0.001; L+PBS vs. L+dBcAMP: *, *p* < 0.05, ***p < 0.001). No significant increase in the BDNF content in sham control (S+PBS) or sham dBcAMP (S+dBcAMP) treated groups.

BDNF level was significantly increased in the dBcAMP treated groups compared to control groups at 7th post injury day in stab wound injury (*p* < 0.001, Figure 8A), and in excitotoxic lesion (*p* < 0.01–0.001, Figure 8B). There were no significant changes in BDNF contents in sham groups (S+PBS vs. S+ dBcAMP).

### Effect of BDNF-receptor inhibitor on neurodegeneration

**Figure 9:**
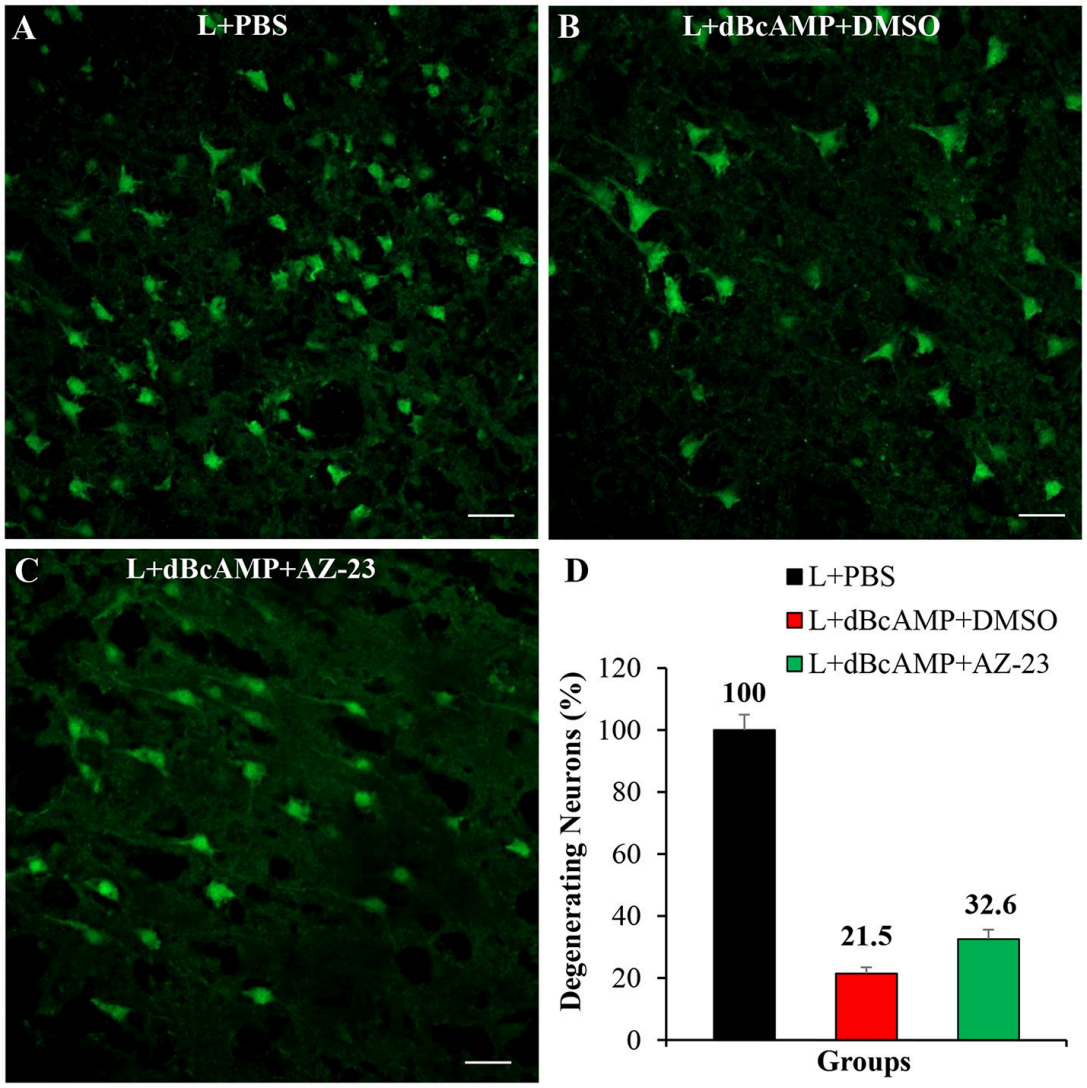
Photomicrograph of Fluoro-Jade B stained sections of the brain, 4 days after lesion (L)/lesion+treatment showing degenerating neurons in L+PBS **(A)**, L+dBcAMP+DMSO **(B)**, and L+dBcAMP+AZ-23 **(C)** groups (Stab wound injury). Note the increased number of degenerating neurons in AZ-23 (BDNF-receptor inhibitor) treated group. Scale bar = 30 μm. Graphs showing the number of degenerating neurons (% of L+PBS) around the stab wound injury site **(D)**. Number of degenerating neurons in dBcAMP+AZ-23 treated group increased compared to L+PBS group.

Treatment with BDNF-receptor inhibitor, AZ-23, significantly increased the degeneration of neurons around the lesion site. In mice that were treated with dBcAMP alone, the percentage of degenerating neurons was 21% of control mice group. However, in mice that were treated with dBcAMP and AZ-23 the percentage of degenerating neurons was 32% of control mice group (i.e., increase in the percentage of degenerating neurons in BDNF receptor inhibitor treated group) (Figure 9).

## Discussions

Repair after brain injury remains a significant challenge. Extensive neuronal degeneration after injury results in prominent disruption of the functional activity of the brain. Using agents that inhibit neuronal degeneration is a promising approach to limit the degenerating neurons and functional defect accompanying brain trauma. In this study we demonstrated that the number of degenerating neurons were significantly decreased in dBcAMP-treated animals in both types of brain injuries. A previous study has demonstrated that dBcAMP has a neuroprotective effect against ecotoxicity (Mena et al., 1995). Other investigators used dBcAMP to induce differentiation of neural stem cells into neurons (Kim et al., 2002; Tojima et al., 2003; Kume et al., 2008). In addition, agents that increase intracellular cAMP have been shown to be neuroprotective in the acute phase of spinal cord injury (Nakao, 1998; Bretzner et al., 2010). Moreover, downstream pathways of cAMP are important for survival of newly generated neurons during development (Jagasia et al., 2009). dBcAMP may also have beneficial effect with respect to promoting axonal regeneration (Hannila and Filbin, 2008) and neurites outgrowth (Cai et al., 2002; Gao et al., 2003).

The main type of cells that are affected in brain injuries are neurons, but glial cells may be indirect targets of brain injury, because they support the function of neurons. Among the glial cells are astrocytes that regulate neuronal survival and functions. Previous studies have shown that dBcAMP induced astrogliosis in neural tissue cultures (Abd-El-Basset, 2000; Aras et al., 2012). In the present study we demonstrated that in both types of brain injuries there were activation of both astrocytes and microglia and both cells increased significantly with dBcAMP treatment. In addition, it was also found that dBcAMP stimulated the expression of GFAP.

In brain injuries both activated astrocytes and microglia play dual role in promoting beneficial and detrimental effect on neurons by producing neuroprotective factors or cytotoxic mediators and proinflammatory cytokines respectively depending upon the functional response (Chio et al., 2015; Karve et al., 2016; Kumar et al., 2017). In this study induction of astrogliosis and activation of microglia may account for the neuroprotection in both types of injuries. One mechanism is the increase in the expression of BDNF as demonstrated in this study. We demonstrated that BDNF receptor (Trk kinase) inhibitor increases the degenerating neurons, which suggests the neuroprotective role of BDNF. However, the number of degenerating neurons does not reach the control level indicating that there are other factors beside the BDNF may be involved. In the future, identification other factors promoted by dBcAMP that account for neuroprotection may facilitate our understanding of the role of dBcAMP in neuronal protection. Previous studies have demonstrated BDNF immunoreactivity in astrocytes, microglia and endothelial cells after brain injury, suggesting its neuroprotective role (Béjot et al., 2011) and the overexpression of the BDNF in hippocampal astrocytes promotes local neurogenesis and elicits anxiolytic-like activities (Quesseveur et al., 2013). Activated astrocytes not only overexpress BDNF but also trap extracellular BDNF which promotes migration of neuronal precursors toward the ischemic striatum (Grade et al., 2013). In addition, BDNF plays an active role in clearance of GABA from synaptic and extrasynaptic sites by enhancing GABA transport in cortical astrocytes and in this way influence neuronal excitability (Vaz et al., 2011).

Previous studies have shown that cAMP modulates the secretion of BDNF in developing airway smooth muscle (Thompson et al., 2015) and influences the neurites length (Xu et al., 2012) and regeneration of adult CNS (Spencer and Filbin, 2004). Other studies have demonstrated that the activation of cAMP response element binding protein (CREB) pathway and elevation of neurotrophic nerve growth factor (NGF) and BDNF protect against neuronal amyloid toxicity (Counts and Mufson, 2010) and has an important role in modulating mood (Nair and Vaidya, 2006). In addition, dBcAMP potentiated or induced the survival promoting effects of BDNF on cultured cerebellar granule cells (Frank et al., 2000). The critical role of cAMP levels in determining the growth cone's response to BDNF gradient (Song et al., 1997) and in modulating a variety of neuronal and behavioral objects have been shown (Ueda and Wu, 2012).

As BDNF does not cross blood brain barrier (BBB) (Chodobski et al., 2011; Xiang et al., 2014), researchers are challenged to develop BDNF gene and protein delivery methods to the CNS (Nagahara and Tuszynski, 2011; Géral et al., 2013), or injection of BDNF-expressing mesenchymal stem cells (Gransee et al., 2015). Molecules that increases endogenous BDNF or conversion of proBDNF to mature BDNF are important as a novel treatment options. In this study we used dBcAMP to promote the secretion of BDNF and enhance neuronal survival in brain injuries.

## Conclusions

This study indicates that dBcAMP induces astrogliosis and promotes the survival of cortical neurons in stab wound and hippocampal neurons in excitotoxic (kainic acid) brain injuries. dBcAMP activates astrocytes and microglia and both activated astrocytes and microglia contribute to the neuronal survival by increasing the secretion of BDNF as its receptor inhibition affected (decreased) the neuronal protection. Hence dBcAMP may be considered for use as therapeutic agent for treatment of traumatic brain injuries.

## Author contributions

All authors listed have made a substantial, direct and intellectual contribution to the work, and approved it for publication.

### Conflict of interest statement

The authors declare that the research was conducted in the absence of any commercial or financial relationships that could be construed as a potential conflict of interest.
